# Comprehensive In Vitro and In Silico Analysis of Antimicrobial and Insecticidal Properties of Essential Oil of *Myrtus communis* L. from Algeria

**DOI:** 10.3390/ijms26104754

**Published:** 2025-05-15

**Authors:** Ghozlane Barboucha, Noureddine Rahim, Amina Bramki, Houssem Boulebd, Anna Andolfi, Khaoula Boulacheb, Amina Boulacel, Maria Michela Salvatore, Marco Masi

**Affiliations:** 1 Biotechnology Laboratory, Higher National School of Biotechnology Taoufik Khaznadar, Nouveau Pôle Universitaire Ali Mendjeli, Constantine 25100, Algeria; g.barb@ensbiotech.edu.dz (G.B.); n.rahim@ensbiotech.edu.dz (N.R.); boulacheb.kh@gmail.com (K.B.); aminaboulacel@gmail.com (A.B.); 2 Laboratory of Bio Engineering, Higher National School of Biotechnology Taoufik Khaznadar, Nouveau Pôle Universitaire Ali Mendjeli, Constantine 25100, Algeria; a.bramki@ensbiotech.edu.dz; 3 Laboratory of Synthesis of Molecules with Biological Interest, Department of Chemistry, Faculty of Exact Sciences, University Frères Mentouri Constantine 1, Constantine 25017, Algeria; boulebd.houssem@umc.edu.dz; 4 Department of Chemical Sciences, University of Naples Federico II, 80126 Naples, Italy; andolfi@unina.it; 5 Department of Veterinary Medicine and Animal Production, University of Naples Federico II, 80137 Naples, Italy; mariamichela.salvatore@unina.it

**Keywords:** *Myrtus communis*, essential oil, antibacterial, antifungal, insecticidal, DFT calculations, molecular docking

## Abstract

This study investigated the phytochemical composition and biological activities of *Myrtus communis* essential oil (EO) from Algeria, focusing on its antimicrobial, antifungal, and insecticidal properties using in vitro and in silico approaches. Gas chromatography–mass spectrometry (GC-MS) analysis identified myrtenyl acetate (57.58%), 1,8-cineole (17.82%), and α-terpineol (6.82%) as the major constituents. *M. communis* EO exhibited significant antibacterial activity, particularly against *Staphylococcus aureus* (13.00 ± 0.70 mm) and *Salmonella typhimurium* (13.00 ± 1.50 mm), with moderate inhibition of *Bacillus subtilis* (10 ± 1.00 mm) and *Escherichia coli* (9.00 ± 0.70 mm), while *Pseudomonas aeruginosa* showed resistance. The antifungal activity was notable against *Fusarium oxysporum* (16.50 ± 0.50 mm), *Aspergillus fumigatus* (11.00 ± 1.00 mm), and *Penicillium* sp. (9.00 ± 0.60 mm) but ineffective against *Aspergillus niger*. Insecticidal activity against *Tribolium castaneum* was evaluated using contact toxicity, fumigation toxicity, and repellent activity assays. The EO demonstrated potent insecticidal effects, with an LC_50_ value of 0.029 µL/insect for contact toxicity and 162.85 µL/L air for fumigation after 96 h. Additionally, the EO exhibited strong repellent activity, achieving 99.44% repellency at a concentration of 0.23 mg/cm^2^ after 24 h. Density functional theory (DFT) calculations provided insights into the molecular geometry and electronic properties of the key bioactive compounds. Molecular docking studies evaluated their binding affinities to bacterial enzymes (*DNA gyrase*, *dihydrofolate reductase6*, and *Gyrase B*) and insecticidal targets (*acetylcholinesterase*), revealing strong interactions, particularly for geranyl acetate and methyleugenol. These findings highlight *M. communis* EO as a promising natural antimicrobial and insecticidal agent, with potential applications in plant protection and biopesticide development.

## 1. Introduction

In recent years, natural bioactive compounds, particularly essential oils (EOs), have garnered significant attention due to their broad applications in medicine, agriculture, and the food industry [1]. These volatile secondary metabolites, synthesized by aromatic plants, are known for their distinct chemical compositions and wide range of biological activities, including antimicrobial, antioxidant, anti-inflammatory, and insecticidal properties [2,3,4]. EOs are increasingly recognized as valuable alternatives to synthetic pesticides, which pose challenges such as resistance development, environmental toxicity, and potential risks to human health [5,6,7]. As a result, the search for more sustainable and eco-friendly pest control solutions has intensified, with EOs playing a key role in integrated pest management strategies [8].

The growing resistance of pests and pathogens to synthetic chemical pesticides highlights the need for natural alternatives that are both effective and safer for humans and the environment [9,10]. EOs, with their broad-spectrum biological activities, have shown great promise as natural biopesticides, offering effective pest management in both agricultural and domestic settings while minimizing ecological impact [5].

*Myrtus communis* L. (common myrtle), an aromatic evergreen shrub native to the Mediterranean region, has long been used in traditional herbal medicine for treating respiratory and digestive disorders [11,12]. The EO extracted from its leaves contains bioactive terpenes such as 1,8-cineole, α-pinene, myrtenyl acetate, and linalool, which contribute to its antimicrobial, insecticidal, and antioxidant properties [13]. These attributes have prompted growing interest in *M. communis* EO as a potential biopesticide, particularly for managing stored-product pests and pathogens, which are major concerns in agriculture and the food industry [14].

Despite increasing interest, the chemical composition and biological activity of *M. communis* EO from specific regions, such as Mila province in Algeria, remain poorly understood. To address this gap, the present study aims to characterize the chemical profile of *M. communis* EO using gas chromatography–mass spectrometry (GC-MS) and evaluate its biological activity against a range of pathogens, including bacterial and fungal strains, as well as the insect *Tribolium castaneum*, a common pest in stored products. To the best of our knowledge, this study represents the first investigation into the contact toxicity and repellent properties of *M. communis* EO against *T. castaneum*.

## 2. Results and Discussion

### 2.1. M. communis Essential Oil Yield

The essential oil of *M. communis* leaves was obtained with a yield of 0.67% (*w*/*w*), which is in agreement with the results obtained by Wannes et al. [15] and Mohamadi et al. [16], who reported yields of 0.61% and 0.68 ± 0.6%, respectively, while Mimica-Dukić et al. [17] recorded slightly higher yields ranging from 0.72% to 0.81%. In contrast, Satrani et al. [18], using a different extraction technique, achieved a lower yield of 0.3 to 0.4%, emphasizing the impact of the extraction method on oil recovery. These differences highlight the influence of multiple factors, including material quality, environmental factors, or extraction parameters, on essential oil yield and the importance of optimizing extraction conditions for improved recovery [19].

### 2.2. Composition of M. communis Essential Oil

**Figure 1:**
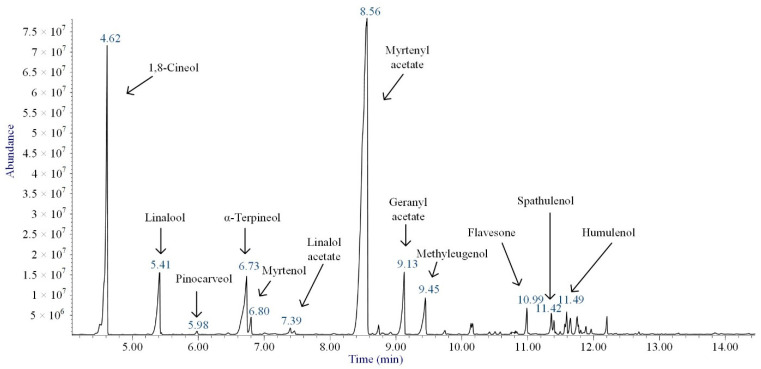
Annotated total ion current chromatogram (TICC) from GC-MS analysis of the essential oil extracted from *M. communis* leaves.

The EO of *M. communis* leaves obtained via hydrodistillation has been analyzed by GC-MS (Figure 1). The most common components usually found in myrtle EO were present in the analyzed sample. Table 1 reports the retention indices and area percentage of the components identified. Several papers have reported that factors, mainly related to genetics and the environment, affect the variability in the chemical composition of EO extracted from plants [20,21], and the EO composition of myrtle seems to be quite variable [22]. As previously reported [23], the content of the bicyclic monoterpene myrtenyl acetate is important to differentiate essential oils of myrtle leaves representing the main constituent of our sample (57.58%), followed by the oxygenated monoterpenes 1,8-cineol (17.83%), α-terpineol (6.83%), and linalool (5.46%). Other components were present in a percentage less than 5%, including geranyl acetate (4.57%), methyleugenol (3.08%), flavesone (1.16%), spathulenol (1.14%), and humulenol (0.86%), pinocarveol (0.20%), myrtenol (1.01%), and linalool acetate (0.29%).

### 2.3. Antibacterial Activity

The results of the antibacterial activity showed that *M. communis* EO was able to inhibit the growth of all bacterial strains except *P. aeruginosa*, with inhibition zone diameters that were more or less significant compared to gentamicin. The different diameters of the inhibition zones obtained are illustrated in Table 2.

The most significant antibacterial effects were observed against *S. aureus* and *S. typhimurium*, with diameters of 13 ± 0.70 and 13 ± 1.5 mm, respectively, followed by moderate effects against *B. subtilis* and *E. coli*, with diameters of 10 ± 1.00 and 9 ± 0.70 mm, respectively. However, it showed a slightly weak effect against *K. pneumoniae*, with a diameter of 7.5 ± 0.50 mm. Our results are consistent with those of Falleh et al. [24], who found that *M. communis* EO exerts a moderate inhibitory effect against 14 bacterial strains, with inhibition zone diameters ranging from 6 to 18 ± 0.7 mm. Similarly, it was demonstrated that *M. communis* EO exhibits moderate inhibitory activity against four tested strains: *Acinetobacter baumannii*, *S. aureus*, *S. epidermidis*, and *K. pneumoniae*, where the largest inhibition zone (18 mm) was observed with *S. aureus* [25]. *M. communis* EO from nineteen localities along the Algerian coastline turned out to be effective against *S. aureus*, *Salmonella enterica, Proteus mirabilis*, and *E. coli* [16]. Furthermore, Ouedrhiri et al. [26], showed that *M. communis* EO exhibited a moderate effect against *S. aureus*, *B. cereus*, and *E. coli*, with inhibition zones measuring between 11.5 ± 0.5 and 17.33 ± 1.52 mm. In contrast, it showed no activity against *P. aeruginosa*. The GC-MS analysis showed the presence of bioactive compounds, primarily myrtenyl acetate, 1,8-cineole, α-terpineol, linalool, geranyl acetate, and methyleugenol, all of which are well known for their antibacterial effects. Myrtenyl acetate, for instance, has been shown to inhibit the growth of several bacterial strains, including *E. coli*, *Listeria monocytogenes*, *Pectobacterium carotovorum*, *P. aeruginosa*, and *S. aureus* [27]. Another major compound, 1,8-cineole, also plays a crucial role in antibacterial activity. It exerts a bactericidal effect by increasing bacterial membrane permeability, which leads to the leakage of proteins and nucleic acids [28]. It also induces oxidative stress, contributing to the death of bacterial cells. Supporting this, Barhouchi et al. [29] reported through in vitro studies and molecular docking investigations that 1,8-cineole is likely the primary phytochemical responsible for the antibacterial activity of *M. communis* EO. Similarly, α-terpineol has been found to disrupt bacterial membrane function, ultimately leading to cell death [30]. Likewise, linalool exhibits potent antibacterial activity against *P. aeruginosa* by damaging its cell membrane, increasing its permeability, and disrupting the respiratory chain, which together result in bacterial cell death [31]. Moreover, geranyl acetate, an ester derived from geraniol, has been reported to possess significant antimicrobial properties [32]. Additionally, methyleugenol has also been shown to exhibit strong antimicrobial activity against a broad spectrum of bacterial strains [33], reinforcing the potential of these bioactive compounds as effective antibacterial agents.

### 2.4. Antifungal Activity

The antifungal activity results obtained by the disc diffusion method are presented in Table 3.

The antifungal test showed that the EO of *M. communis* exhibited a moderate effect compared to nystatin against the four strains (*A. fumigatus*, *Penicillium* sp., *C. albicans*, and *F. oxysporum*), where the highest effect was observed against *F. oxysporum*, with a diameter of 16.5 ± 0.5 mm, while slightly weaker effects were noted against *A. fumigatus*, *Penicillium* sp., and *C. albicans*, with diameters ranging from 8.0 ± 0.6 to 11.0 ± 1.0 mm. However, no antifungal effect was observed against *A. niger*. In fact, the antifungal activity of *M. communis* EO has been proven by several researchers, including Cannas et al. [34], who reported that *M. communis* EO was effective against various *Candida* species, even at low concentrations. In a previous study [35], two *M. communis* EOs showed varying degrees of inhibition against all tested fungi, such as *C. albicans*, *A. niger*, and *A. fumigatus.* It was also demonstrated that *M. communis* EO was able to inhibit the growth of several fungal species from the genera *Aspergillus* spp., *Fusarium* spp., and *Penicillium* spp., with inhibition zone diameters ranging from 8 to 30 mm [36]. In fact, the antimicrobial potential of EOs is mainly attributed to their chemical constituents and functional groups [37]. As revealed by the GC-MS analysis, the main compounds in *M. communis* EO are myrtenyl acetate, 1,8-cineole, α-terpineol, linalool, geranyl acetate, and methyleugenol. While most of these compounds are well known for their antifungal properties, the antifungal activity of myrtenyl acetate remains largely unexplored. To the best of our knowledge, no studies have specifically investigated its effects. On the other hand, 1,8-cineole has been reported to exhibit notable antifungal potential. Ivanov et al. [38] observed that this compound inhibited the growth of *Candida glabrata* and *Candida parapsilosis*, with a minimum inhibitory concentration (MIC) of 2 mg/mL. In line with this, Ghazi Mirsaid et al. [39] described its antidermatophytic properties, with MIC values ranging from 0.78 to 25 mg/mL. Additionally, its broad antimicrobial and antiviral effects have been demonstrated, reinforcing its relevance in the treatment of various infectious diseases [40]. Similarly, α-terpineol has been reported to inhibit both mycelial growth and spore germination of *A. niger* [41]. Likewise, linalool, a monoterpene found in various plant genera, has been described as having fungicidal effects against *C. albicans*, including fluconazole-resistant strains, with an MIC of 256 µg/mL [42]. Furthermore, geranyl acetate has been documented for its antifungal activity and potential synergistic effects when combined with fluconazole [43]. As for methyleugenol, Joshi [33] reported its antimicrobial activity against *A. niger*, *A. fumigatus*, and *P. chrysogenum*, further emphasizing its potential role as an antifungal agent.

### 2.5. Insecticidal Activity

#### 2.5.1. Contact Toxicity

The contact topical toxicity of *M. communis* EO against *T. castaneum* adults is presented in Table 4 and Table 5.

The mortality percentages of *T. castaneum* increased progressively with higher concentrations of *M. communis* EO, demonstrating a clear concentration-dependent response (Table 4). Mortality rates ranged from 3.33% at the lowest concentration (0.020 μL/insect) to 90.0% at the highest concentration (0.045 μL/insect) after 96 h. Significant differences in mortality were observed across concentrations and exposure times, as indicated by the one-way ANOVA results: at 24 h (F = 51.83, *p* < 0.05), 48 h (F = 150.73, *p* < 0.05), 72 h (F = 60.30, *p* < 0.05) and 96 h (F = 96.85, *p* < 0.05).

At 0.045 μL/insect, the EO consistently showed the highest toxicity, achieving mortality rates of 76.67% within 24 h and reaching 90.0% by 96 h. In contrast, at lower concentrations (0.020 μL/insect), the mortality rates remained below 14% over the same period.

The probit analysis revealed that the lethal concentrations of *M. communis* EO required to achieve 50% (LC_50_) and 90% (LC_90_) mortality in *T. castaneum* adults decreased over time, demonstrating a time-dependent increase in EO efficacy (Table 5). Specifically, the chronic LC_50_ and LC_90_ values were determined to be 0.029 and 0.049 μL/insect, respectively, further confirming the concentration-dependent toxicity of the EO.

To the best of our knowledge, this study is the first to investigate the contact toxicity of *M. communis* EO against *T. castaneum*. While research on the insecticidal properties of *M. communis* EO remains limited, existing studies highlight its significant bioactivity against various agricultural and medical pests. For instance, Yezli et al. [44] reported an LC₅₀ value of 17.50 μL/mL against *Culex pipiens*, while Benddine et al. [45] observed 88.13% mortality in *Rhopalosiphum maidis* at a concentration of 6 μL/mL. Similarly, an LC_50_ of 0.42 mg/cm^2^ was documented for *Sitophilus oryzae* [46], and an LC_50_ of 6.1 mg/mL was documented for *Anopheles stephensi*, a major malaria vector [47].

In our study, myrtenyl acetate (57.6%) and 1,8-cineole (17.8%) were identified as the predominant constituents of MEO, likely contributing to its insecticidal activity. Liska et al. [48] demonstrated that 1,8-cineole, extracted from *Eucalyptus* species, caused complete mortality in *T. castaneum* at a dose of 0.2 μL/insect. Similarly, Yildirim et al. [49] reported 99% mortality in *S. zeamais* at a concentration of 10 μL of 1,8-cineole.

Although the insecticidal activity of myrtenyl acetate has not been specifically investigated, its structural similarity to other monoterpenes suggests potential efficacy. For example, Yildirim et al. [49] evaluated the toxicity of various monoterpenes against *S. zeamais*, reporting 54.55% mortality for bornyl acetate, 43.43% for geranyl acetate, and 72.73% for neryl acetate. Given these results, myrtenyl acetate may also exhibit significant insecticidal activity, warranting further investigation.

#### 2.5.2. Fumigant Toxicity

Table 6 presents the mortality rates of *T. castaneum* exposed to different concentrations of *M. communis* EO via the fumigation method over four exposure periods (24, 48, 72, and 96 h). The results demonstrate a clear concentration- and time-dependent toxicity of the EO.

At the lowest concentration (100 μL/L air), mortality was minimal, with only 6.66% observed at 72 h and 10% at 96 h (Table 6). However, mortality increased significantly with higher concentrations of *M. communis* EO. For instance, at 500 μL/L air (the highest concentration tested), mortality reached 96.7% at 24 h and 100% at 48 h. Similarly, at 400 μL/L air, complete mortality (100%) was achieved within 72 h, and at 200 μL/L air, mortality rose from 13.33% at 24 h to 76.66% at 96 h.

The statistical analysis (one-way ANOVA) confirmed significant differences in mortality between the various concentrations across all exposure times (all *p*-values < 0.005), highlighting the important role of both concentration and exposure time in the observed effects.

These results emphasize the potent insecticidal activity of *M. communis* EO by fumigation, with higher concentrations and longer exposure times leading to increased mortality. The EO was highly effective, achieving 100% mortality at 400 μL/L and 500 μL/L air concentrations, positioning it as a promising candidate for pest control.

Probit analysis further supported the time-dependent increase in efficacy, with LC_50_ values decreasing from 295.79 µL/L air at 24 h to 162.85 µL/L air at 96 h and LC_90_ values dropping from 437.55 µL/L air to 275.01 µL/L air over the same period (Table 7). These findings underscore the potential of *M. communis* EO as a highly effective fumigant.

Numerous studies have demonstrated the fumigant properties of *M. communis* EO against stored-product pests. For instance, Khani & Basavand [50] reported high fumigant toxicity of *M. communis* EO from Iran, with an LC₅₀ value of 261 μL/L air against *Tribolium confusum*, and an even stronger effect against *Callosobruchus maculatus*, a major pest of cowpeas, with an LC₅₀ of 9.50 μL/L air. Similarly, Senfi et al. [51] determined LC_50_ and LC_95_ values of *M. communis* EO against *T. castaneum* to be 243.78 μL/L and 685.85 μL/L, respectively. However, geographical variations appear to influence its efficacy, with a lower fumigant activity for *M. communis* EO from Tunisia, with LC_50_ and LC_95_ values of 357.67 μL/L and 530.69 μL/L, respectively [52].

The biological activity of *M. communis* EO is largely attributed to its major volatile constituents, particularly myrtenyl acetate and 1,8-cineole. Fassbinder et al. [53] demonstrated that myrtenyl acetate exhibits significant fumigant toxicity, with an LC_50_ value of 0.07 µg/cm^3^ against *Varroa destructor*, confirming its potent acaricidal properties. Kheloul et al. [54] reported that 1,8-cineole was responsible for the fumigant toxicity of *E. globulus* EO against *T. castaneum*, with an LC₅₀ value of 149.685 µL/L air. Furthermore, Sharma and Tiwari [55] found that 1,8-cineole exhibited strong toxicity against *S. oryzae*, achieving 100% mortality within 24 h at a concentration of 200 μL/L air. According to Cao et al. [56], linalool, another key volatile compound in *M. communis* EO, exhibited potent fumigant toxicity against *T. castaneum*, with an LC_50_ value of 45.96 μg/adult.

Given these findings, the pronounced fumigant toxicity observed for *M. communis* EO in this study can be attributed to the presence of these bioactive compounds. Myrtenyl acetate and 1,8-cineole likely act independently or in synergy to exert their insecticidal effects, reinforcing the potential of *M. communis* EO as an alternative fumigant. This natural fumigant offers several advantages over conventional chemical treatments, which are often associated with environmental persistence, toxicity to non-target organisms, and risks to human health. Moreover, as resistance to synthetic fumigants such as phosphine and pyrethroids continues to rise [57], EO-based alternatives, with their complex chemical composition, may serve as a sustainable solution to pest management by reducing the likelihood of resistance development.

#### 2.5.3. Repellency Activity

The repellent activity of the EO against *T. castaneum* was evaluated at different concentrations (2, 4, 6, and 8 µL) over 24 h. Results showed a dose-dependent effect, with repellence percentages ranging from 85.55% (2 µL) to 99.44% (8 µL) (Table 8). Higher concentrations exhibited prolonged repellence, with 8 µL maintaining 100% repellence up to 12 h and 96.66% at 24 h. All tested concentrations fell within Class V (highly repellent, PR ≥ 80.1%), highlighting the strong repellent potential of *M. communis* EO.

In this study, we evaluated the repellent activity of *M. communis* EO against *T. castaneum*, providing, to the best of our knowledge, the first report on its efficacy against this pest.

Beyond its efficacy against *T. castaneum*, *M. communis* EO has demonstrated repellent activity against a range of other insect pests. For instance, Salehi et al. [58] observed a 61.3% repellency rate against *Ephestia kuehniella*, a major stored-product pest, at the highest tested concentration (2.00 µL/L air). Similarly, Tavassoli et al. [59] reported significant repellent activity of *M. communis* EO against *A. stephensi*, with an LC_50_ value of 0.111 mg/cm^2^. These findings are consistent with our results and further underscore the broad-spectrum repellent potential of *M. communis* EO across diverse insect species. The repellent properties of *M. communis* EO are likely attributable to its rich composition of monoterpenes, well-documented herbivore-induced volatiles with insect-repellent properties [60]. These compounds play a key role in plant defense by deterring herbivory and minimizing insect damage [61]. For instance, 1,8-cineole exhibits high repellent activity against *T. castaneum*, achieving 90% repellency at a concentration of 0.0943 mg/cm^2^ after 8 h of exposure [62]. Similarly, Cao et al. [56] reported a 58% repellency rate against *T. castaneum* when exposed to linalool at 0.067 mg/cm^2^ after 4 h.

Our findings suggest that *M. communis* EO is a promising natural alternative to synthetic repellents for managing *T. castaneum* infestations. Its high repellency at relatively low concentrations makes it an environmentally friendly option for integrated pest management strategies. Future research should focus on identifying the specific bioactive compounds responsible for its repellent activity, optimizing formulations for practical applications, and assessing its efficacy under field conditions.

#### 2.5.4. In Silico Investigations

**Figure 2:**
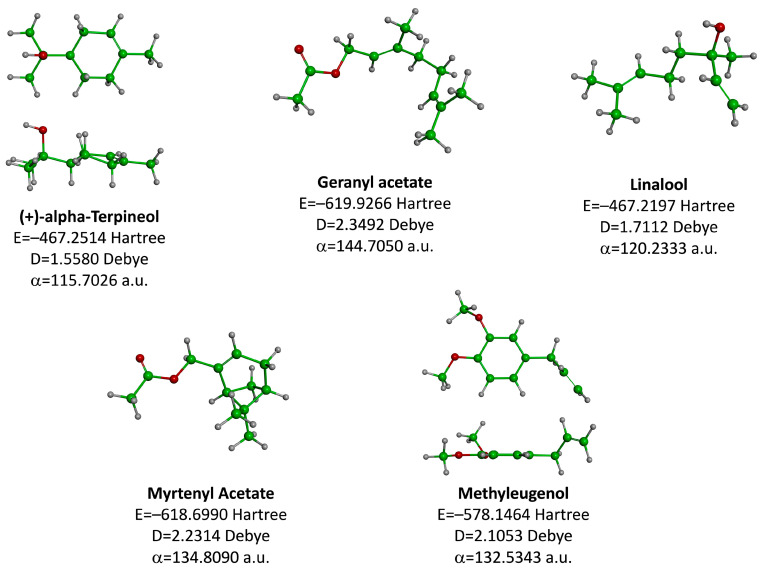
Computed molecular geometry of the primary constituents present in *M. communis* EO: linalool, (+)-α-terpineol, myrtenyl acetate, geranyl acetate, and methyleugenol.

The molecular geometry of the primary constituents present in *M. communis* EO, including linalool, α-terpineol, myrtenyl acetate, geranyl acetate, and methyleugenol, was determined using DFT calculations at the B3LYP/6-311G (d,p) level. The structure of 1,8-cineole, another major constituent of the EO, was investigated in our previous study [63]. The optimized geometries, dipole moments (D), and polarizability (α) of these compounds are illustrated in Figure 2.

The D values, which indicate the polarity of the molecules, ranged from 1.5580 Debye for (+)-α-Terpineol to 2.3492 Debye for Geranyl acetate. The higher dipole moments of geranyl acetate (2.3492 Debye), myrtenyl acetate (2.2314 Debye), and methyleugenol (2.1053 Debye) suggest that these molecules may have stronger intermolecular interactions compared to linalool (1.7112 Debye) and (+)-α-terpineol (1.5580 Debye). Additionally, the α values, which measure the ability of a molecule to be distorted by an external electric field, varied among the compounds. Geranyl acetate exhibited the highest polarizability (144.7050 a.u.), followed by myrtenyl acetate (134.8090 a.u.) and methyleugenol (132.5343 a.u.). Linalool (120.2333 a.u.) and (+)-α-terpineol (115.7026 a.u.) had comparatively lower polarizability values. These molecular properties play a crucial role in defining the physicochemical behavior of these EO constituents, which can influence their biological activity, solubility, and interactions with other molecules.

To explore the potential antimicrobial and insecticidal properties of *M. communis* EO, molecular docking studies were conducted on its key constituents against four target enzymes: *E. coli* DNA gyrase (PDB: 6RKU), *E. coli* dihydrofolate reductase (DHFR, PDB: 4DFR), *E. coli* Gyrase B (PDB: 6F86), and *Drosophila melanogaster* acetylcholinesterase (AChE). These enzymes play critical roles in bacterial survival and insect neurotransmission, making them prime targets for antimicrobial and insecticidal agents.

DNA gyrase is a bacterial enzyme responsible for introducing negative supercoils into DNA, a process essential for maintaining DNA topology and genome integrity. Given its unique function, it is a key target for various antibacterial drugs. Similarly, DHFR is an essential enzyme involved in folate metabolism, catalyzing the conversion of dihydrofolate to tetrahydrofolate, a necessary step for DNA synthesis. Inhibition of DHFR disrupts bacterial replication, making it a valuable target for antimicrobial agents such as trimethoprim.

Gyrase B, another critical enzyme in bacterial DNA replication, alleviates torsional stress during DNA synthesis by facilitating the supercoiling of DNA. Its inhibition can effectively halt bacterial proliferation. On the other hand, acetylcholinesterase (AChE) is an enzyme essential for nerve function in insects. It breaks down acetylcholine at synapses, ensuring proper signal transmission. Inhibiting AChE disrupts neurotransmission, leading to neuromuscular dysfunction, paralysis, and eventual insect death. This mode of action is widely recognized in the development of insecticidal compounds [64].

The results shown in Table 9 reveal that, compared to the native ligands, all tested compounds exhibited weaker binding energies, indicating lower inhibitory potential. Among the EO constituents, Geranyl acetate displayed the strongest binding interactions across all targets, with binding energies of −3.77 kcal/mol (DNA gyrase), −3.55 kcal/mol (DHFR), −3.30 kcal/mol (Gyrase B), and −3.85 kcal/mol (dmAChE). Similarly, methyleugenol also demonstrated favorable interactions, particularly with DNA gyrase (−3.73 kcal/mol) and Gyrase B (−3.33 kcal/mol). Linalool and (+)-α-terpineol showed comparatively weaker binding affinities, especially against Gyrase B (−2.56 and −2.76 kcal/mol, respectively), suggesting a lower potential for bacterial inhibition. Myrtenyl acetate exhibited moderate interactions, particularly with DHFR (−3.60 kcal/mol) and dmAChE (−3.52 kcal/mol).

**Figure 3:**
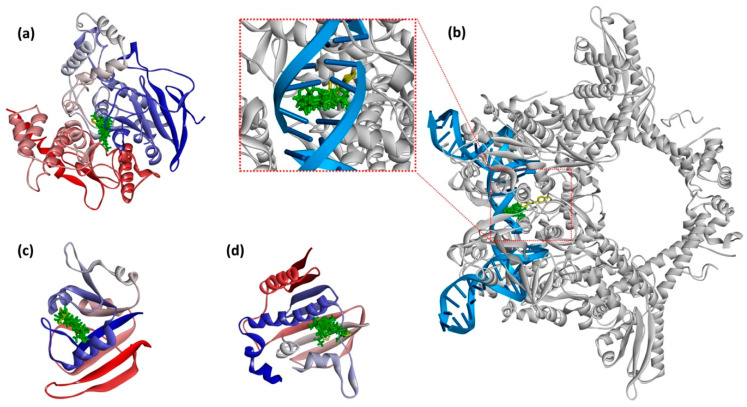
Superimposition of the docked poses of all investigated compounds into the active site of dmAChE (**a**), DNA Gyrase (**b**), DHFR (**c**), and GyraseB (**d**).

**Figure 4:**
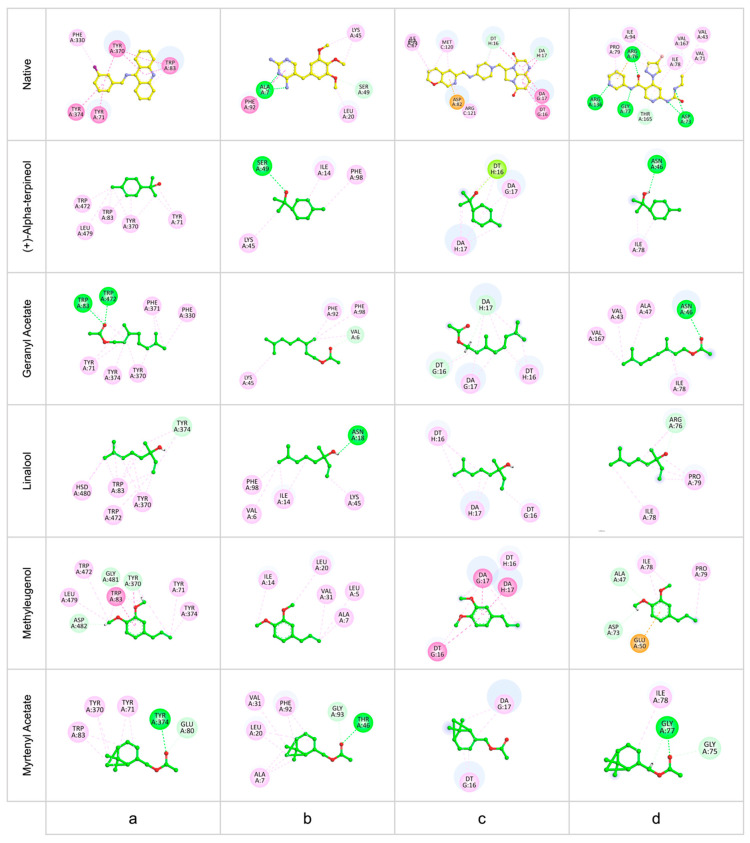
Two-dimensional representation of the interaction modes of all investigated compounds into the active site of dmAChE (**a**), DHFR (**b**), DNA Gyrase (**c**), and GyraseB (**d**).

The superposition of the most stable poses of the investigated compounds into the active sites of the target enzymes is illustrated in Figure 3, while 2D representations of the interaction modes of all the compounds are illustrated in Figure 4. The 2D interaction diagrams provide essential information on the molecular interactions stabilizing ligand binding and highlight the key residues involved in the formation of hydrogen bonds and hydrophobic interactions.

Interactions between EO compounds and critical active site residues are similar to those of native ligands. In dmAChE, interactions with TYR374 and TRP83 are frequently observed, suggesting that these residues play a central role in stabilizing ligands within the enzyme’s active site. Similarly, in DHFR, PHE92 and LEU20 are involved in hydrophobic interactions with compounds such as geranyl acetate, methyleugenolet, and myrtenyl acetate, indicating that these residues are essential for ligand binding. In DNA gyrase, all compounds interacted with DTG16 and DAG17, key nucleotides in the enzyme’s active site, demonstrating their ability to mimic native ligand interactions. Finally, in Gyrase B, ILE78 and PRO79 are residues frequently involved in ligand stabilization, suggesting that these compounds could act as competitive inhibitors by occupying the same binding pockets as the native ligand.

Hydrogen bonds, depicted in green in the figure, play a crucial role in stabilizing enzyme–ligand complexes and were observed for most of the studied compounds. Notably, myrtenyl Acetate formed hydrogen bonds with dmAChE, DHFR, and Gyrase B. In contrast, methyleugenol did not exhibit any hydrogen bonding interactions with the investigated enzymes.

Despite the moderate binding energies of the studied compounds compared to the native ligands, their favorable interaction patterns with key residues in the active sites of the enzymes suggest their potential as inhibitors. These interactions may, in part, explain the antimicrobial and insecticidal activities of *M. communis* EO, highlighting the potential of these compounds in targeting essential enzymatic functions.

## 3. Materials and Methods

### 3.1. Plant Material and Essential Oil Extraction

Fresh adult leaves of *Myrtus communis* were collected in March 2023 in the Hamala region, Mila province, northeast Algeria (36°34′18″ N, 6°20′24″ E). The collected plant was washed with distilled water to remove any debris and then dried in a well-ventilated and shaded area at room temperature for 20 days. After drying, the leaves were milled using a mechanical grinder. Essential oil extraction was performed using a Clevenger-type apparatus via hydrodistillation at 120 °C for 4 h [65]. The obtained EO was stored in an amber glass vial at 4 °C until further use in GC-MS characterization and biological activities. The essential oil yield (%) was determined by calculating the ratio of the extracted oil weight to the weight of the dried *M. communis* leaves, according to the formula:

$$Yield\;\left(\%\right)=\frac{weight\;of\;collected\;essential\;oil}{dry\;weight\;of\;extracted\;plant\;}\times 100$$

### 3.2. GC-MS Analysis

Essential oils were analyzed using an Agilent 6850 GC instrument (Milan, Italy) coupled to an Agilent 5973 Inert MS. For the separation, an Agilent J&W HP-5ms Ultra Inert GC capillary column (stationary phase: (5%-phenyl)-methylpolysiloxane; length: 30 m; ID: 0.25 mm; film thickness: 0.25 µm) was housed in the GC oven. The following oven GC temperature program was employed: 70 °C for 1 min; 10 °C/min until the column temperature reached 170 °C; 30 °C/min until the column temperature reached 280 °C; 280 °C for 8 min. The solvent delay was set to 4 min. Helium was employed as carrier gas at a flow rate of 1 mL/min, and the GC injector was set at 250 °C, split ratio 1/10. Measurements were conducted under electron impact (EI) ionization (70 eV) in full scan mode (*m*/*z* 35–550) at a frequency of 3.9 Hz. The EI ion source and quadrupole mass filter temperatures were kept, respectively, at 200 and 250 °C. Essential oils were identified by comparing their EI mass spectra at 70 eV with mass spectra collected in the NIST 20 mass spectral library (available online: https://www.nist.gov/srd/nist-standard-reference-database-1a accessed on 17 March 2025). The identification was further supported by the Kovats retention index (RI) calculated for each analyte by the Kovats equation, using the standard *n*-alkane mixture in the range of C7–C40 (Sigma-Aldrich, Saint Louis, MO, USA) analyzed under the same conditions.

### 3.3. Antibacterial Activity

The antibacterial activity of the *M. communis* EO was evaluated using disc diffusion technique against ATCC test bacteria (American Type Culture Collection), which are *Staphylococcus aureus* (ATCC 25923), *Bacillus subtilis* (ATCC 6633), *Escherichia coli* (ATCC 25922), *Pseudomonas aeruginosa* (ATCC 27853), *Salmonella typhimurium* (ATCC 14028), and *Klebsiella pneumoniae* (ATCC 700603). Bacterial suspensions were obtained from cultures incubated at 37 °C for 24 h. The cell density of each suspension was standardized by diluting it in sterile physiological water until reaching a turbidity corresponding to 0.5 McFarland [66]. Sterile disks of 6 mm in diameter of Whatman paper were soaked with 10 μL of sample (1 mg of EO dissolved in 10 µL of dimethyl sulfoxide (DMSO)) and placed on the surface of Mueller–Hinton agar plates inoculated with the test organisms. The plates were kept at 4 °C for 2 h to allow the diffusion of bioactive compounds and then incubated at 37 °C for 24 h. Gentamicin (10 µg) was used as the positive control, while DMSO served as the negative control [67].

### 3.4. Antifungal Activity

The antifungal activity of *M. communis* EO at the same concentration was evaluated using the disc diffusion method previously described against *Aspergillus niger* (MH109542), *Aspergillus fumigatus* (MH109539), *Penicillium* sp., *Fusarium oxysporum*, and *Candida albicans.* The yeast *C. albicans* was seeded on Sabouraud medium and then incubated at 37 °C for 24 h. Following this, the density of the suspension was adjusted to 0.5 McFarland in sterile physiological water [68]. Conversely, the molds were subcultured on PDA (Potato Dextrose Agar) medium and incubated at 28 °C for 14 days. Dense suspensions of spores were obtained by culture scraping after the addition of physiological water. The suspensions were diluted until reaching an absorbance of 0.15 to 0.2 at 650 nm. These suspensions were diluted to 1/10. The test was performed on Sabouraud medium. The yeast *C. albicans* was incubated at 37 °C for 24 to 48 h, while the molds were incubated at 28 °C for 48 to 72 h. For this test, nystatin was used as a positive control, whereas DMSO was used as a negative control [63].

### 3.5. Insecticidal Activity

#### 3.5.1. Test Insect Culture

The *Tribolium castaneum* specimens used in this study were reared under controlled laboratory conditions on a wheat flour-based medium. Insects were maintained in plastic containers (25 cm in height, 12 cm in width) filled with 200 g of wheat flour supplemented with 5% (*w*/*w*) brewer’s yeast. To allow for oviposition, adult insects (approximately 4 mm × 1.5 mm) were transferred to fresh containers every five days. Cultures were routinely monitored to ensure age uniformity. Rearing conditions were maintained at 27 ± 2 °C and 80 ± 5% relative humidity. Only two-week-old adults were selected for bioassays, which were conducted under the same environmental conditions as the rearing setup.

#### 3.5.2. Contact Toxicity Assay

The contact toxicity assay of the EO against *T. castaneum* was conducted in glass Petri dishes (9 cm in diameter) according to the method described by Barboucha et al. [63]. Six concentrations were prepared by diluting 20, 25, 30, 35, 40, and 45 µL of EO in 1 mL of acetone, resulting in final doses of 0.02, 0.025, 0.03, 0.035, 0.04, and 0.045 µL per insect. A volume of 1 μL from each dilution was carefully applied directly onto the thorax of each test insect. A control was treated with only 1 μL of acetone.

For each concentration and control, ten insects were used, and the experiment was performed in triplicate. After treatment, both the treated and control insects were placed in Petri dishes containing 10 g of sterile crushed wheat seeds. The number of dead insects was recorded daily after each 24 h period, and the percentage of mortality was calculated and corrected using the Abbott formula [69]:

$$Calculated\;mortality\;\left(\%\right)=\left(1-\frac{n\;in\;T\;after\;treatment}{n\;in\;Co\;after\;treatment}\right)\times 100$$

where *n* represents the number of insects, *T* denotes the treated group, and *Co* refers to the control group.

#### 3.5.3. Fumigant Toxicity Assay

The fumigant toxicity of *M. communis* EO was evaluated using a vapor-phase bioassay, following the methodology described by Wanna and Bozdoğan [62]. The experiments were conducted in 60 mL Falcon tubes (5 × 4 cm) with screw caps. To facilitate EO volatilization, small cotton pieces were attached to the inner side of the caps using cotton threads and treated with 5, 10, 15, 20, and 25 µL of EO, corresponding to air concentrations of 100, 200, 300, 400, and 500 µL/L, respectively. Ten adults of *T. castaneum* were introduced into each tube, which was then securely sealed to ensure exposure to EO vapors. Each concentration was tested in triplicate, alongside a control group (cotton without EO). Mortality was assessed at 24, 48, 72, and 96 h, with insects considered dead if they remained immobile with no leg or antennal movements detected.

#### 3.5.4. Repellency Assay

The repellent activity of the EO against *T. castaneum* was assessed using a zone-choice bioassay, following the method outlined by McDonald et al. [70]. The assay was conducted in glass Petri dishes (9 cm in diameter). Four concentrations were prepared by diluting 2, 4, 6, and 8 µL of EO in 1 mL of acetone. Whatman No. 1 filter papers (9 cm in diameter) were bisected, with one half treated with 0.5 mL of the EO solution and the other with 0.5 mL of acetone as a control. Both halves were left to air-dry for 15 min to allow complete solvent evaporation before being arranged adjacently inside the Petri dish. Ten adult insects were placed at the center of each dish, and the experiment was conducted in triplicate for each concentration. Insect distribution between the treated and control halves was recorded at 30 min, 4 h, 6 h, 8 h, 12 h, and 24 h post-exposure. The percentage of repulsion (PR) was determined for each concentration and at each exposure time using the formula of McDonald et al. [70]:

$$RP=\frac{\left(NC-NT\right)}{\left(NC+NT\right)}\times 100$$

where *PR* represents the percentage of repellence, *NC* is the number of test insects in the control half disc, and *NT* represents the number of test insects in the treated half.

The percentage of repulsion (PR) was categorized into six repellent classes based on the scale established by McDonald et al. [70]. The classification was as follows: class 0 (PR < 0.1%)—non-repellent; class I (PR: 0.1–20.0%)—very weak repellent; class II (PR: 20.1–40.0%)—weak repellent; class III (PR: 40.1–60.0%)—moderately repellent; class IV (PR: 60.1–80.0%)—repellent; and class V (PR: 80.1–100.0%)—highly repellent.

### 3.6. DFT Calculations

The molecular geometry of the primary constituents present in *M. communis* EO was elucidated through density functional theory (DFT) calculations. These computational analyses were carried out employing the Gaussian 09 software [71], utilizing the B3LYP functional combined with the 6-311G (d,p) basis set. To ensure the reliability of the calculated ground states, the absence of imaginary frequencies was rigorously verified, confirming the stability and accuracy of the optimized structures.

### 3.7. Molecular Docking

The molecular geometry of the investigated molecules was determined using DFT calculations. The structural coordinates of the target enzymes, including *E. coli* DNA gyrase (PDB code: 6RKU), *E. coli* dihydrofolate reductase (DHFR, PDB code: 4DFR), *E. coli* Gyrase B (PDB code: 6F86), and *Drosophila melanogaster* acetylcholinesterase (dmAChE, PDB code: 6XYU), were retrieved from the Protein Data Bank. Prior to docking studies, ligands, water molecules, heteroatoms, and co-crystallized solvents were removed from the protein structures. Partial charges and hydrogen atoms were then added to the proteins using LePro software (available online: http://www.lephar.com, accessed on 3 January 2025). The docking search space was defined as a 25 Å cubic grid with 1 Å spacing, centered on the active site of each protein. Molecular docking simulations were conducted using LeDock software (http://www.lephar.com, accessed on 3 January 2025), and visual representations of the results were generated using BIOVIA Discovery Studio. The docking protocol was validated by comparing the crystallographic data of native ligands with theoretical docking results, yielding a root mean square deviation (RMSD) value of less than 2 Å, confirming the reliability of the docking approach.

### 3.8. Statistical Analysis

All results are presented as mean ± standard error (SE). Differences between variables were assessed using one-way ANOVA followed by Tukey’s HSD post hoc test. Probit analysis was conducted to determine LC_50_ and LC_90_ values, along with their 95% fiducial confidence limits. Statistical analyses were performed using SPSS software (version 25.0, IBM-SPSS Inc., Chicago, IL, USA), with significance set at *p* < 0.05.

## 4. Conclusions

The increasing resistance of pathogens and insect pests to conventional pesticides, along with growing environmental and health concerns, has intensified the search for safer and more sustainable alternatives. In this context, *Myrtus communis* essential oil (EO) emerges as a promising natural agent for antimicrobial and insecticidal applications. Phytochemical analysis revealed a high concentration of bioactive monoterpenes, including myrtenyl acetate, 1,8-cineole, and α-terpineol, which contribute to its biological efficacy. Experimental studies demonstrated significant antibacterial and antifungal activity, particularly against *S. aureus*, *S. typhimurium*, and *F. oxysporum*, reinforcing its potential as a natural antimicrobial agent. Additionally, the EO exhibited potent insecticidal effects against *T. castaneum* in a dose-dependent manner, with strong contact toxicity, fumigation toxicity, and repellency properties.

Molecular docking studies provided mechanistic insights, revealing strong interactions between EO constituents and key biological targets, such as bacterial enzymes (*DNA gyrase*, *dihydrofolate reductase*) and insect acetylcholinesterase, supporting its mode of action. Furthermore, density functional theory (DFT) calculations confirmed the favorable molecular properties of the major compounds. However, despite its promising potential, challenges such as volatility and stability may limit the direct application of *M. communis* EO in real-world agricultural settings. To enhance its effectiveness, innovative formulations, such as nanoemulsions and encapsulation techniques, should be explored.

It is important to note that all experiments in this study were conducted under controlled laboratory conditions. Therefore, further research is required to assess the practical efficacy of *M. communis* EO in field applications, including its long-term stability, persistence, and potential effects on non-target organisms. These investigations will be crucial for validating its role as a sustainable alternative for integrated pest management and crop protection strategies.

## Figures and Tables

**Table 1:** Phytochemical constituents contained in the EO extracted from Algerian *M. communis.* RT represents the retention time. RI_exp_ and RI_NIST20_ represent, respectively, Kovats programmed temperature retention indices evaluated in this work (on a 5% phenylmethylpolysiloxane) and gathered from NIST20 mass spectral library (for identical or similar) stationary phases. %ΔRI is the percent difference between RI_exp_ and RI_NIST20_.

Compound	Chemical Structure	RI_exp_	RI_NIST20_	%ΔRI	Area %
1,8-Cineol	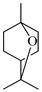	1034	1032	0.19	17.83
Linalool	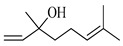	1112	1103	0.82	5.46
Pinocarveol	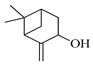	1154	1139	1.32	0.20
α-Terpineol	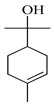	1211	1194	1.42	6.83
Myrtenol	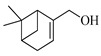	1216	1202	1.16	1.01
Linalol acetate	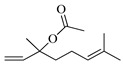	1260	1258	0.16	0.29
Myrtenyl acetate	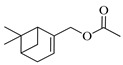	1348	1335	0.97	57.58
Geranyl acetate	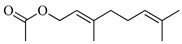	1382	1384	−0.14	4.57
Methyleugenol	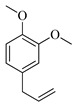	1418	1412	0.42	3.08
Flavesone	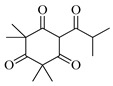	1546	1546	0.00	1.16
Spathulenol	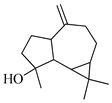	1616	1620	−0.25	1.14
Humulenol	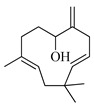	1666	1650	0.97	0.86

**Table 2:** Antibacterial activity of *M. communis* EO compared to an antibiotic.

	Diameter of Inhibition Zone in mm					
	*S. aureus*	*B. subtilis*	*E. coli*	*P. aeruginosa*	*S. typhimurium*	*K. pneumoniae*
EO	13 ± 0.70 ^bc^	10 ± 1.00 ^cd^	9 ± 0.70 ^d^	-	13 ± 1.5 ^bc^	7.5 ± 0.50 ^d^
Gentamicin	17.5± 0.50 ^a^	18.75 ± 0.25 ^a^	11 ± 0.30 ^cd^	16.5 ± 0.50 ^ab^	15.5 ± 0.40 ^ab^	10.5 ± 0.50 ^cd^

(-): No activity. Superscript values with different letters within the same row are significantly different (*p* < 0.05).

**Table 3:** In vitro analysis of the antifungal activity of *M. communis* EO with disc diffusion method.

	Diameter of Inhibition Zone in mm				
	*A. niger*	*A. fumigatus*	*Penicillium* sp.	*C. albicans*	*F. oxysporum*
EO	-	11 ± 1.0 ^e^	9 ± 0.60 ^e^	8 ± 0.6 ^e^	16.5 ± 0.5 ^d^
Nystatin	24.5 ± 0.25 ^c^	32.5 ± 0.51 ^b^	39.5 ± 0.70 ^a^	22 ± 0.51 ^c^	33 ± 1.4 ^b^

(-): No activity. Superscript values with different letters within the same row are significantly different (*p* < 0.05).

**Table 4:** Corrected percent mortality (means ± SE) of *T. castaneum* adults after application of different concentrations of *M. communis* EO at different time intervals.

Concentration (μL/Insect)		Mortality (%)			
		24 h	48 h	72 h	96 h
0.020		3.33 ± 3.33 ^d^	10.0 ± 0.0 ^d^	13.33 ± 3.33 ^d^	13.33 ± 3.33 ^e^
0.025		26.45 ± 1.13 ^c^	30.0 ± 0.0 ^c^	36.65 ± 4.13 ^c^	43.45 ± 4.22 ^d^
0.030		26.61 ± 3.11 ^c^	30.0 ± 0.0 ^c^	46.67 ± 3.21 ^bc^	53.33 ± 2.33 ^cd^
0.035		50.0 ± 5.77 ^b^	53.33 ± 3.03 ^b^	53.33 ± 3.03 ^b^	60.0 ± 0.0 ^c^
0.040		66.66 ± 2.43 ^ab^	76.66 ± 1.31 ^a^	76.66 ± 1.31 ^a^	76.66 ± 1.31 ^b^
0.045		76.67 ± 3.33 ^a^	83.33 ± 3.33 ^a^	83.33 ± 3.33 ^a^	90.0 ± 0.0 ^a^
One-way ANOVA	F value	51.83	150.73	60.30	96.85
	*p*-value	<0.005	<0.005	<0.005	<0.005

Means within the same column followed by same letter are not significantly different (*p* < 0.05).

**Table 5:** Lethal concentrations (LC_50_ and LC_90_) on contact toxicity of *M. communis* EO against *T. castaneum* at different exposure times.

Exposure Time (h)	LC_50_ ^a^ (μL/Insect)	LC_90_ ^a^ (μL/Insect)	Slope ± SEM ^b^	Chi-Square (χ^2^)	df
24	0.035 (0.033–0.036)	0.055 (0.051–0.061)	6.46 ± 0.55	9.24	4
48	0.033 (0.030–0.036)	0.052 (0.045–0.070)	6.32 ± 0.52	10.10	4
72	0.031 (0.029–0.032)	0.053 0.048–0.059)	5.52 ± 0.50	5.73	4
96	0.029 (0.028–0.031)	0.049 (0.045–0.054)	5.81 ± 0.50	8.28	4

LC: lethal concentration. ^a^ Values in the bracket represent lower and upper confidence limit; ^b^ SEM: mean standard error.

**Table 6:** Percent mortality of *T. castaneum* after exposure to different concentrations of *M. communis* EO applied by fumigation method. Means within the same column followed by same letter are not significantly different (*p* < 0.05).

Concentration		24 h	48 h	72 h	96 h
100 μL/L air		0	0	6.66 ± 3.33 ^c^	10 ± 0.0 ^d^
200 μL/L air		13.33 ± 2.33 ^c^	66.66 ± 3.33 ^b^	73.34 ± 3.13 ^b^	76.16 ± 1.96 ^c^
300 μL/L air		43.43 ± 1.86 ^b^	76.67 ± 2.55 ^b^	83.21 ± 4.11 ^b^	86.65 ± 4.33 ^b^
400 μL/L air		86.66 ± 3.42 ^a^	96.33 ± 3.02 ^a^	100 ^a^	100 ^a^
500 μL/L air		96.70 ± 2.21 ^a^	100 ^a^	100 ^a^	100 ^a^
One-way ANOVA	F value	207.88	245.50	223.67	315.75
	*p*-value	<0.005	<0.005	<0.005	<0.005

Means within the same column followed by same letter are not significantly different (*p* < 0.05).

**Table 7:** Lethal concentrations of *M. communis* EO against *T. castaneum* adults at different exposure times.

Exposure Time (h)	LC_50_ ^a^ (µL/Liter Air)	LC_90_ ^a^ (µL/Liter Air)	Slope ± SEM ^b^	Chi-Square (χ^2^)	df
24	295.79 (281.35–310.02)	437.55 (410.81–473.88)	7.54 ± 0.60	4.95	3
48	197.73 (110.50–262.01)	321.67 (244.78–725.51)	6.06 ± 0.48	22.12	3
72	172.86 (116.94–221.47)	288.36 (224.72–495.45)	5.77 ± 0.44	15.22	3
96	162.85 (118.18–202.99)	275.01 (219.12–424.68)	5.63 ± 0.43	11.46	3

LC: lethal concentration. ^a^ Values in the bracket represent lower and upper confidence limit; ^b^ SEM: mean standard error.

**Table 8:** Repellent activity (%) of *M. communis* EO against *T. castaneum* at different exposure times and concentrations.

Concentration		Repellence (%)						Mean Repellence	Repellent Class
		30 min	4 h	6 h	8h	12 h	24 h		
2 µL (0.06 mg/cm^2^)		86.70 ± 3.10 ^a^	100 ^a^	90 ± 5.77 ^a^	86.66 ± 3.33 ^b^	80 ^b^	70 ± 5.77 ^b^	85.55 ± 1.47 ^c^	V
4 µL (0.11 mg/cm^2^)		96.67 ± 3.33 ^a^	96.67 ± 3.33 ^a^	93.33 ± 3.22 ^a^	93.33 ± 3.22 ^ab^	90 ± 5.80 ^ab^	83.33 ± 6.66 ^ab^	92.22 ± 2.42 ^bc^	V
6 µL (0.17 mg/cm^2^)		100 ^a^	100 ^a^	100 ^a^	96.66 ± 3.33 ^ab^	96.66 ± 3.33 ^a^	86.66 ± 3.33 ^ab^	96.67 ± 0.96 ^ab^	V
8 µL (0.23 mg/cm^2^)		100 ^a^	100 ^a^	100 ^a^	100 ^a^	100 ^a^	96.66 ± 3.33 ^a^	99.44 ± 0.56 ^a^	V
One-way ANOVA	F value	7.17	1.00	2.25	3.89	7.00	4.85	15.86	
	*p*-value	0.0123	0.441	0.160	0.055	0.013	0.033	0.01	

Means within the same column followed by same letter are not significantly different (*p* < 0.05).

**Table 9:** Docking binding energies in kcal/mol of *M. communis* EO with DNA gyrase (PDB code: 6RKU), DHFR (PDB code: 4DFR), GyraseB (PDB code: 6F86), and dmAChE (PDB code: 6XYU) enzymes.

Compound	Docking Binding Energy in kcal/mol			
	DNA Gyrase	DHFR	GyraseB	dmAChE
(+)-α-terpineol	−3.22	−2.92	−2.76	−3.10
Geranyl acetate	−3.77	−3.55	−3.30	−3.85
Linalool	−2.95	−3.09	−2.56	−3.04
Methyleugenol	−3.73	−3.36	−3.33	−3.69
Myrtenyl acetate	−2.88	−3.60	−3.28	−3.52
Native ligand	−7.28	−5.71	−6.92	−7.14

## Data Availability

The data presented in this study are available on request from the corresponding author.

## References

[1] Bolouri P., Salami R., Kouhi S., Kordi M., Asgari B., Hadian J., Astatkie T. (2022). Applications of essential oils and plant extracts in different industries. Moleculas.

[2] Angane M., Swift S., Huang K., Butts C.A., Quek S.Y. (2022). Essential oils and their major components: An updated review on antimicrobial activities, mechanism of action and their potential application in the food industry. Foods.

[3] de Sousa D.P., de Assis Oliveira F., Arcanjo D.D.R., da Fonsêca D.V., Duarte A.B.S., de Oliveira Barbosa C., Ong T.P., Brocksom T.J. (2024). Essential Oils: Chemistry and Pharmacological Activities—Part II. Biomedicines.

[4] Rahim N., Derabli C., Bramki A., Mahdjoub S., Rup-jacques S., Barboucha G., Hesse S., Boulebd H. (2025). Evaluating the multifaceted bioactivity of *Syzygium aromaticum* essential oil: The central role of eugenol Evaluating the multifaceted bioactivity of *Syzygium aromaticum* essential oil: The. Turk. J. Biol. Vol..

[5] Assadpour E., Can Karaça A., Fasamanesh M., Mahdavi S.A., Shariat-Alavi M., Feng J., Kharazmi M.S., Rehman A., Jafari S. (2024). Application of essential oils as natural biopesticides; recent advances. Crit. Rev. Food Sci. Nutr..

[6] Ngegba P.M., Cui G., Khalid M.Z., Zhong G. (2022). Use of botanical pesticides in agriculture as an alternative to synthetic pesticides. Agriculture.

[7] Prakash B., Singh P.P., Gupta V., Raghuvanshi T.S. (2024). Essential oils as green promising alternatives to chemical preservatives for agri-food products: New insight into molecular mechanism, toxicity assessment, and safety profile. Food Chem. Toxicol..

[8] Quinn E., Ben-Simchon E., Gorelick J., Oka Y., Frenkel O., Sionov E., Kostyukovsky M., Dudai N., Shimshoni J., Zilkah S. (2024). Examination of genetic lines of *Myrtus communis* as potential sources of organic agricultural pest control agents. Heliyon.

[9] Khursheed A., Rather M.A., Jain V., Wani A.R., Rasool S., Nazir R., Malik N.A., Majid S.A. (2022). Plant based natural products as potential ecofriendly and safer biopesticides: A comprehensive overview of their advantages over conventional pesticides, limitations and regulatory aspects. Microb. Pathog..

[10] Reyes-Ávila A., López-Ruiz R., Egea González F.J., Romero-González R., Garrido Frenich A. (2024). Chemistry and development of bioinsecticides for safe and sustainable use. Curr. Opin. Environ. Sci. Health.

[11] Ali K., Ullah F., Khan N., Rahman I.U., Ullah S., Khan W., Ali M., Uddin N., Nisar M. (2017). Ethnobotanical and ecological study of *Myrtus communis* (L.) in Bajaur agency (FATA) Khyber-Pakhtunkhwa, Pakistan. J. Biodivers. Environ. Sci..

[12] Al-Snafi A.E., Teibo J.O., Shaheen H.M., Akinfe O.A., Teibo T.K.A., Emieseimokumo N., Elfiky M.M., Al-kuraishy H.M., Al-Garbeeb A.I., Alexiou A. (2024). The therapeutic value of *Myrtus communis* L.: An updated review. Naunyn-Schmiedeberg’s Arch. Pharmacol..

[13] Hennia A., Nemmiche S., Dandlen S., Miguel M.G. (2019). *Myrtus communis* essential oils: Insecticidal, antioxidant and antimicrobial activities: A review. J. Essent. Oil Res..

[14] Vega E.N., González-Zamorano L., Cebadera E., Barros L., da Silveira T.F.F., Vidal-Diez de Ulzurrun G., Tardío J., Lázaro A., Cámara M., Fernández-Ruíz V. (2025). Wild *Myrtus communis* L. fruit by-product as a promising source of a new natural food colourant: Optimization of the extraction process and chemical characterization. Foods.

[15] Wannes W.A., Mhamdi B., Sriti J., Jemia M.B., Ouchikh O., Hamdaoui G., Kchouk M.E., Marzouk B. (2010). Antioxidant activities of the essential oils and methanol extracts from myrtle (*Myrtus communis* var. *italica* L.) leaf, stem and flower. Food Chem. Toxicol..

[16] Mohamadi Y., Lograda T., Ramdani M., Figueredo G., Chalard P. (2021). Chemical composition and antimicrobial activity of *Myrtus Communis* essential oils from Algeria. Biodiversitas.

[17] Mimica-Dukic N., Bugarin D., Grbovic S., Mitic-Culafic D., Vukovic-Gacic B., Orcic D., Jovin E., Couladis M. (2010). Essential oil of *Myrtus communis* L. As a potential antioxidant and antimutagenic agents. Molecules.

[18] Satrani B., Farah A., Talbi M. (2006). Effet de la distillation fractionnée sur la composition chimique et l’activité antimicrobienne des huiles essentielles du Myrte (*Myrtus communis* L.) du Maroc. Acta Bot. Gall..

[19] Mugao L. (2024). Factors influencing yield, chemical composition and efficacy of essential oils. Int. J. Multidiscip. Res. Growth Eval..

[20] Laftouhi A., Eloutassi N., Ech-Chihbi E., Rais Z., Abdellaoui A., Taleb A., Beniken M., Nafidi H.A., Salamatullah A.M., Bourhia M. (2023). The impact of environmental stress on the secondary metabolites and the chemical compositions of the essential oils from some medicinal plants used as food supplements. Sustainability.

[21] Mkaddem M.G., Zrig A., Ben Abdallah M., Romdhane M., Okla M.K., Al-Hashimi A., Alwase Y.A., Hegab M.Y., Madany M.M.Y., Hassan A.H.A. (2022). Variation of the chemical composition of essential oils and total phenols content in natural populations of *Marrubium vulgare* L.. Plants.

[22] Vafadar Shoshtari Z., Rahimmalek M., Sabzalian M.R., Hosseini H. (2017). Essential oil and bioactive compounds variation in myrtle (*Myrtus communis* L.) as affected by seasonal variation and salt stress. Chem. Biodivers..

[23] Bradesi P., Tomi F., Casanova J., Costa J., Bemardini A.F. (1997). Chemical composition of myrtle leaf essential oil from Corsica (France). J. Essent. Oil Res..

[24] Falleh H., Ben Jemaa M., Djebali K., Abid S., Saada M., Ksouri R. (2019). Application of the mixture design for optimum antimicrobial activity: Combined treatment of *Syzygium aromaticum*, *Cinnamomum zeylanicum*, *Myrtus communis*, and *Lavandula stoechas* essential oils against *Escherichia coli*. J. Food Process. Preserv..

[25] El Hartiti H., El Mostaphi A., Barrahi M., Ali A.B., Chahboun N., Amiyare R., Zarrouk A., Bourkhiss B., Ouhssine M. (2020). Chemical composition and antibacterial activity of the essential oil of *Myrtus communis* leaves. Karbala Int. J. Mod. Sci..

[26] Ouedrhiri W., Mechchate H., Moja S., Baudino S., Saleh A., Al Kamaly O.M., Grafov A., Greche H. (2022). Optimized antibacterial effects in a designed mixture of essential oils of *Myrtus communis*, *Artemisia herba-alba* and *Thymus serpyllum* for wide range of applications. Foods.

[27] Caputo L., Capozzolo F., Amato G., De Feo V., Fratianni F., Vivenzio G., Nazzaro F. (2022). Chemical composition, antibiofilm, cytotoxic, and anti-acetylcholinesterase activities of *Myrtus communis* L. leaves essential oil. BMC Complement. Med. Ther..

[28] Moo C.L., Osman M.A., Yang S.K., Yap W.S., Ismail S., Lim S.H.E., Chong C.M., Lai K.S. (2021). Antimicrobial activity and mode of action of 1,8-cineol against carbapenemase-producing *Klebsiella pneumoniae*. Sci. Rep..

[29] Barhouchi B., Menacer R., Bouchkioua S., Mansour A., Belattar N. (2023). Compounds from myrtle flowers as antibacterial agents and SARS-CoV-2 inhibitors: In-vitro and molecular docking studies. Arab. J. Chem..

[30] Yang X., Zhao S., Deng Y., Xu W., Wang Z., Wang W., Lv R., Liu D. (2023). Antibacterial activity and mechanisms of α-terpineol against foodborne pathogenic bacteria. Appl. Microbiol. Biotechnol..

[31] Liu X., Cai J., Chen H., Zhong Q., Hou Y., Chen W., Chen W. (2020). Antibacterial activity and mechanism of linalool against *Pseudomonas aeruginosa*. Microb. Pathog..

[32] Celuppi L.C.M., Capelezzo A.P., Cima L.B., Zeferino R.C.F., Zanetti M., Riella H.G., Fiori M.A. (2022). Antimicrobial cellulose acetate films by incorporation of geranyl acetate for active food packaging application. Res. Soc. Dev..

[33] Joshi R. (2013). Chemical composition, in vitro antimicrobial and antioxidant activities of the essential oils of *Ocimum gratissimum*, *O. sanctum* and their major constituents. Indian J. Pharm. Sci..

[34] Cannas S., Molicotti P., Ruggeri M., Cubeddu M., Sanguinetti M., Marongiu B., Zanetti S. (2013). Antimycotic activity of *Myrtus communis* L. towards *Candida* spp. from isolates. J. Infect. Dev. Ctries..

[35] Bouzabata A., Cabral C., Gonçalves M.J., Cruz M.T., Bighelli A., Cavaleiro C., Casanova J., Tomi F., Salgueiro L. (2015). *Myrtus communis* L. as source of a bioactive and safe essential oil. Food Chem. Toxicol..

[36] Siddique S., Perveen Z., Nawaz S., Shahzad K., Ali Z. (2015). Chemical composition and antimicrobial activities of essential oils of six species from family Myrtaceae. J. Essent. Oil-Bear. Plants.

[37] Burt S. (2004). Essential oils: Their antibacterial properties and potential applications in foods—A review. Int. J. Food Microbiol..

[38] Ivanov M., Kannan A., Stojković D.S., Glamočlija J., Calhelha R.C., Ferreira I.C.F.R., Sanglard D., Soković M. (2021). Camphor and eucalyptol—Anticandidal spectrum, antivirulence effect, efflux pumps interference and cytotoxicity. Int. J. Mol. Sci..

[39] Ghazi Mirsaid R., Falahati M., Farahyar S., Ghasemi Z., Roudbary M., Mahmoudi S. (2024). In vitro antifungal activity of eucalyptol and its interaction with antifungal drugs against clinical dermatophyte isolates including *Trichophyton indotineae*. Discov. Public Health.

[40] Pries R., Jeschke S., Leichtle A., Bruchhage K.L. (2023). Modes of action of 1,8-cineol in infections and inflammation. Metabolites.

[41] An P., Yang X., Yu J., Qi J., Ren X., Kong Q. (2019). α-Terpineol and terpene-4-ol, the critical components of tea tree oil, exert antifungal activities in vitro and in vivo against *Aspergillus niger* in grapes by inducing morphous damage and metabolic changes of fungus. Food Control.

[42] Medeiros C.I.S., de Sousa M.N.A., Filho G.G.A., Freitas F.O.R., Uchoa D.P.L., Nobre M.S.C., Bezerra A.L.D., Rolim L.A.D.M.M., Morais A.M.B., Nogueira T.B.S.S. (2022). Antifungal activity of linalool against fluconazoleresistant clinical strains of vulvovaginal *Candida albicans* and its predictive mechanism of action. Braz. J. Med. Biol. Res..

[43] Van Zyl R.L., Seatlholo S.T., Van Vuuren S.F., Viljoen A. (2010). Pharmacological interactions of essential oil constituents on the viability of micro-organisms. Nat. Prod. Commun..

[44] Yezli A., Boudjelida H., Arroussi D.E.R. (2024). Components and toxicological effects of *Myrtus communis* L. (Myrtales: Myrtaceae) essential oil against *Mosquito culex pipiens* L. (Diptera: Culicidae). Appl. Ecol. Environ. Res..

[45] Benddine H., Zaid R., Babaali D., Daoudi-Hacini S. (2023). Biological activity of essential oils of *Myrtus communis* (Myrtaceae, Family) and *Foeniculum vulgare* (Apiaceae, Family) on open fields conditions against corn aphids *Rhopalosiphum maidis* (Fitch, 1856) in western Algeria. J. Saudi Soc. Agric. Sci..

[46] Abdelgaleil S.A.M., Badawy M.E.I., Shawir M.S., Mohamed M.I.E. (2015). Chemical composition, fumigant and contact toxicities of essential oils isolated from egyptian plants against the stored grain insects; *Sitophilus oryzae* L. and *Tribolium castaneum* (Herbst). Egypt. J. Biol. Pest Control.

[47] Firooziyan S., Osanloo M., Basseri H.R., Moosa-Kazemi S.H., Mohammadzadeh Hajipirloo H., Amani A., Sedaghat M.M. (2022). Nanoemulsion of *Myrtus communis* essential oil and evaluation of its larvicidal activity against *Anopheles stephensi*. Arab. J. Chem..

[48] Liska A., Rozman V., Kalinovic I., Ivezic M., Balicevic R. (2010). Contact and fumigant activity of 1,8-cineole, eugenol and camphor against *Tribolium castaneum* (Herbst). Julius-Kühn-Archiv.

[49] Yildirim E., Emsen B., Kordali S. (2013). Insecticidal effects of monoterpenes on *Sitophilus zeamais* Motschulsky (Coleoptera: Curculionidae). J. Appl. Bot. Food Qual..

[50] Khani A., Basavand F. (2012). Chemical composition and insecticidal activity of myrtle (*Myrtus communis* L.) essential oil against two stored-product pests. J. Med. Plants By-Prod..

[51] Senfi F., Safaralizadeh M.H., Safavi S.A., Aramideh S. (2014). Fumigant toxicity of Laurus nobilis and *Myrtus communis* essential oils on larvae and adults of the Red flour beetle, *Tribolium castaneum* Herbst (Col.: Tenebrionidae). Arch. Phytopathol. Plant Prot..

[52] Aouadi G., Soltani A., Grami L.K., Abada M.B., Haouel S., Boushih E., Chaanbi M., Elkahoui S., Hajlaoui M.R., Jemâa J.M.B. (2021). Chemical investigations on Algerian Mentha rotundifolia and *Myrtus communis* essential oils and assessment of their insecticidal and antifungal activities. Int. J. Agric. Biol..

[53] Fassbinder C., Grodnitzky J., Coats J. (2002). Monoterpenoids as possible control agents for Varroa destructor. J. Apic. Res..

[54] Kheloul L., Anton S., Bréard D., Kellouche A. (2023). Fumigant toxicity of some essential oils and eucalyptol on different life stages of *Tribolium confusum* (Coleoptera: Tenebrionidae). Bot. Lett..

[55] Sharma J.H., Tiwari S.N. (2022). Fumigant toxicity of alpha-pinene, beta-pinene, eucalyptol, linalool and sabinene against Rice Weevil, *Sitophilus oryzae* (L.). Pantnagar J. Res..

[56] Cao J.Q., Guo S.S., Wang Y., Pang X., Geng Z.F., Du S.S. (2018). Toxicity and repellency of essential oil from *Evodia lenticellata* Huang fruits and its major monoterpenes against three stored-product insects. Ecotoxicol. Environ. Saf..

[57] Sakka M.K., Mavridis K., Papapostolou K.M., Riga M., Vontas J., Athanassiou C.G. (2024). Development, application and evaluation of three novel TaqMan qPCR assays for phosphine resistance monitoring in major stored product pests *Tribolium castaneum* and *Rhyzopertha dominica*. Pest Manag. Sci..

[58] Salehi T., Karimi J., Hasanshahi G., Askarianzadeh A., Abbasipour H., Garjan A.S. (2014). The effect of essential oils from *Laurus nobilis* and *Myrtus commonis* on the adults of mediterranean flour moth, *Ephestia kuehniella* Zeller (Lep.: Pyralidae). J. Essent. Oil-Bear. Plants.

[59] Tavassoli M., Shayeghi M., Abai M.R., Vatandoost H., Khoobdel M., Salari M., Ghaderi A., Rafi F. (2011). Repellency effects of essential oils of myrtle (*Myrtus communis*), Marigold (*Calendula officinalis*) compared with DEET against Anopheles stephensi on human volunteers. Iran. J. Arthropod-Borne Dis..

[60] Lackus N.D., Lackner S., Gershenzon J., Unsicker S.B., Köllner T.G. (2018). The occurrence and formation of monoterpenes in herbivore-damaged poplar roots. Sci. Rep..

[61] Heil M. (2008). Indirect defence via tritrophic interactions. New Phytol..

[62] Wanna R., Bozdoğan H. (2024). Activity of *Rosmarinus officinalis* (Lamiales: Lamiaceae) essential oil and its main constituent, 1,8-cineole, against *Tribolium castaneum* (Coleoptera: Tenebrionidae). J. Entomol. Sci..

[63] Barboucha G., Rahim N., Boulebd H., Bramki A., Andolfi A., Salvatore M.M., Masi M. (2024). Chemical composition, in silico investigations and evaluation of antifungal, antibacterial, insecticidal and repellent activities of *Eucalyptus camaldulensis* Dehn. leaf essential oil from ALGERIA. Plants.

[64] Ryan M.F., Byrne O. (1988). Plant-insect coevolution and inhibition of acetylcholinesterase. J. Chem. Ecol..

[65] Haj Ammar A., Zagrouba F., Romdhane M., Abderrabba M. (2010). Extraction de l’huile essentielle de myrte (*Myrtus communis* L.) provenant de la Tunisie par hydrodistillation. Acta Hortic..

[66] Sehout I., Boulebd H., Boulcina R., Nemouchi S., Bendjeddou L., Bramki A., Merazig H., Debache A. (2021). Synthesis, crystal structure, Hirshfeld surface analysis, biological evaluation, DFT calculations, and in silico ADME analysis of 4-arylidene pyrazolone derivatives as promising antibacterial agents. J. Mol. Struct..

[67] Bramki A., Frahtia M., Jaouani A., Dahimat L., Kacem Chaouche N. (2019). Extraction and preliminary study of antibacterial compounds of three species of *Aspergillus* genus. Asia-Pac. J. Mol. Biol. Biotechnol..

[68] Nemouchi S., Sehout I., Boulebd H., Boulcina R., Bramki A., Bendjeddou L., Benahsene A.H., Debache A., Nemouchi S., Sehout I. (2023). Facile synthesis, crystal structure, hirshfeld surface analysis, DFT calculation and in vitro antifungal evaluation of 4-arylidene-1H-pyrazol-5(4H)-ones. Org. Prep. Proced. Int..

[69] Abbott W.S. (1925). A method of computing the effectiveness of an insecticide. J. Econ. Entomol..

[70] McDonald L.L., Guy R.H., Speirs R.D. (1970). Preliminary evaluation of new candidate materials as toxicants, repellents, and attractants against stored-product insects. Agricultural Research Service.

[71] Frisch M.J., Trucks G.W., Schlegel H.B., Scuseria G.E., Robb M.A., Cheeseman J.R., Scalmani G., Barone V., Mennucci B., Petersson G.A. (2009). Gaussian 09.

